# Smooth Muscle LDL Receptor-Related Protein-1 Deletion Induces Aortic Insufficiency and Promotes Vascular Cardiomyopathy in Mice

**DOI:** 10.1371/journal.pone.0082026

**Published:** 2013-11-29

**Authors:** Joshua E. Basford, Sheryl Koch, Ahmad Anjak, Vivek P. Singh, Eric G. Krause, Nathan Robbins, Neal L. Weintraub, David Y. Hui, Jack Rubinstein

**Affiliations:** 1 Department of Pathology, Metabolic Diseases Institute, University of Cincinnati College of Medicine, Cincinnati, Ohio, United States of America; 2 Department of Internal Medicine, Division of Cardiovascular Diseases, University of Cincinnati College of Medicine, Cincinnati, Ohio, United States of America; 3 Department of Pharmacology and Cell Biophysics, University of Cincinnati College of Medicine, Cincinnati, Ohio, United States of America; 4 Department of Psychiatry and Behavioral Neuroscience, University of Cincinnati College of Medicine, Cincinnati, Ohio, United States of America; Katholieke Universiteit Leuven, Belgium

## Abstract

Valvular disease is common in patients with Marfan syndrome and can lead to cardiomyopathy. However, some patients develop cardiomyopathy in the absence of hemodynamically significant valve dysfunction, suggesting alternative mechanisms of disease progression. Disruption of LDL receptor-related protein-1 (Lrp1) in smooth muscle cells has been shown to cause vascular pathologies similar to Marfan syndrome, with activation of smooth muscle cells, vascular dysfunction and aortic aneurysms. This study used echocardiography and blood pressure monitoring in mouse models to determine whether inactivation of Lrp1 in vascular smooth muscle leads to cardiomyopathy, and if so, whether the mechanism is a consequence of valvular disease. Hemodynamic changes during treatment with captopril were also assessed. Dilation of aortic roots was observed in young Lrp1-knockout mice and progressed as they aged, whereas no significant aortic dilation was detected in wild type littermates. Diastolic blood pressure was lower and pulse pressure higher in Lrp1-knockout mice, which was normalized by treatment with captopril. Aortic dilation was followed by development of aortic insufficiency and subsequent dilated cardiomyopathy due to valvular disease. Thus, smooth muscle cell Lrp1 deficiency results in aortic dilation and insufficiency that causes secondary cardiomyopathy that can be improved by captopril. These findings provide novel insights into mechanisms of cardiomyopathy associated with vascular activation and offer a new model of valvular cardiomyopathy.

## Introduction

Genetic and environmental factors that diminish elasticity and increase vascular stiffness lead to increased risk of atherosclerosis, aortic aneurysm, and vascular dysfunction through several distinct mechanisms [1,2]. Mutations in the fibrillin-1 gene *FBN1* produce vascular defects that are clinically associated with Marfan syndrome [3]. Fibrillin-1 deficiency activates transforming growth factor-β (TGF-β) signaling pathways, leading to elevated collagen synthesis and matrix metalloproteinase-mediated disruption of the elastic fibers in the vessel wall [4], thereby increasing aortic stiffness and decreasing vasoreactivity [5]. Elevated levels of TGF-β have been detected in the vessel wall of patients with Marfan syndrome, in association with aneurysms that most commonly affect the thoracic aorta [6]. Additional reports show that connective tissue growth factor (CTGF), an established downstream mediator of TGFβ-induced fibrogenesis in mesenchymal cells [7], also accumulate in thoracic aortic aneurysms and areas of dissection [8]. 

Another genetic polymorphism associated with increased risk of atherosclerosis, aortic aneurysms, and vascular dysfunction similar to Marfan syndrome is one affecting the *LRP1* gene [9,10]. This gene encodes the LDL receptor related protein-1 (Lrp1) protein that has both cargo endocytosis and cell signal regulatory functions depending on the cell type involved [11]. Proteinases and molecules associated with regulating proteolytic activity represent a majority of Lrp1 ligands and many are relevant to processes that maintain vascular homeostasis. Importantly, Lrp1 has been shown to internalize and degrade CTGF by an array of fibroblast cell types [12]. Further, the expression of Lrp1 in vascular smooth muscle cells mediates TGF-β inhibition of cell proliferation through the Smad protein signaling pathway [13,14]. The importance of smooth muscle cell expression of Lrp1 in vascular homeostasis is best illustrated by observations that smooth muscle-specific inactivation of Lrp1 in mice exaggerates atherosclerosis severity and aortic aneurysm in hypercholesterolemic mice [15]. Smooth muscle Lrp1 deficiency also reduces vascular reactivity, promotes denudation-induced neointimal formation and modulates smooth muscle cell (SMC) phenotype in normolipidemic animals [16]. The vascular protective properties of endogenous Lrp1 have been attributed to its limitation of smooth muscle cell response to platelet-derived growth factor (PDGF) and TGF-β stimulation, with the former contributing to the atherosclerosis phenotype and the latter affecting elastic layer integrity and aneurysm similar to that observed with Marfan syndrome [17]. Consistent with the similar phenotype between Lrp1 deficiency and Marfan syndrome, a recent study showed that LRP1 also protects the vasculature by regulating matrix deposition and limiting protease activity in the vessel wall [18]. 

 The constitutive activation of TGF-β and impaired CTGF clearance and elastogenesis associated with smooth muscle Lrp1 deficiency has been shown to cause aortic dilatation. Thus, smooth muscle Lrp1 deficiency may potentially cause cardiac dysfunction via several mechanisms, such as aortic root dilation leading to aortic insufficiency and the subsequent development of dilated cardiomyopathy secondary to valve disease [19]. While this scenario is clinically observed in patients with Marfan syndrome, there have also been reports of cardiomyopathy developing in these patients in the absence of valve disease [5], raising the possibility that the cardiomyopathy observed with Marfan syndrome is due to genetic defects in the heart instead of an indirect effect as a consequence of vascular abnormalities. However, subclinical valvular disease may be difficult to exclude in these patients, as clinical studies typically do not employ serial imaging over sufficient time periods before and early during the course of developing cardiomyopathy. In the mouse model, cardiac functions were found to be normal in young mice with smooth muscle Lrp1 deficiency [18]. This study was undertaken to test the hypothesis that vascular defects due to smooth muscle Lrp1 deficiency directly lead to cardiomyopathy as the animals progress in age, and to determine if the mechanism is primary or secondary to valvular disease. We sought to evaluate this relationship between aortic insufficiency and cardiomyopathy development with advanced ultrasound imaging that permitted us to longitudinally follow the aorta, aortic valve and left ventricle size and function in individual mouse. As the role of the renin-angiotensin (RAAS) system has been implicated in Marfan syndrome [20,21], we also investigated the potential role of RAAS blockade on hemodynamic responses in these mice.

## Materials and Methods

### Cardiomyocyte Preparation

 Ventricular myocytes were isolated from *smLrp1* ^*+/+*^ and *smLrp1* ^*-/-*^ mice as described [22]. Briefly, hearts were excised from 16 week-old male mice after anesthetization with 70 mg/kg sodium pentobarbital. Hearts were mounted on a Langendorff perfusion apparatus and perfused with Ca^2+^-free Tyrode solution for 6 min at 37°C followed by Tyrode solution containing 0.8 mg/ml collagenase (type B; Boehringer-Mannheim, Indianapolis, IN) and 0.03 mg/ml pronase (Boerhinger-Mannheim) until hearts became flaccid. The tissues were teased apart and the resulting cell suspensions were washed twice in Tyrode buffer and observed microscopically to assess cell purity (>98% myocytes). Cells were lysed in 2x volume of NP40 lysis buffer (Invitrogen, Carlesbad, CA) containing protease inhibitors (Roche, Indianapolis, IN) and assayed with Lrp1 and CTGF immunoblotting. 

### Cardiomyocyte Mechanics Measurement

 Isolation of mouse left ventricular myocytes was carried out as described previously [23]. Briefly, mouse hearts were excised from anesthetized (pentobarbital sodium, 70 mg/kg, i.p.) adult mice, mounted in a Langendorff perfusion apparatus, and perfused with Ca^2+^-free Tyrode solution at 37°C for 3 min. The normal Tyrode solution contained 140 mmol/L NaCl, 4 mmol/L KCl, 1 mmol/L MgCl_2_, 10 mmol/L glucose, and 5 mmol/L HEPES, pH 7.4. Perfusion was then switched to the same solution containing 75 units/ml type 1 collagenase (Worthington), and perfusion continued until the heart became flaccid (~10–15 min). The left ventricular tissue was excised, minced, pipette-dissociated, and filtered through a 240-μm screen. The cell suspension was then sequentially washed in 0.025, 0.1, 0.2 then 1.0 mmol/L Ca^2+^-Tyrode and resuspended in 1.8 mmole/L Ca^2+^-Tyrode for further analysis. The myocyte suspension was placed in a Plexiglas chamber, which was positioned on the stage of an inverted epifluorescence microscope (Nikon Diaphot 200), and perfused with 1.8 mmol/L Ca^2+^-Tyrode solution. Cell shortening was measured at room temperature (22–23°C). The room temperature allowed the myocytes to be stable for up to 2 h with constant pacing. Myocytes were field stimulated to contract by a Grass S5 stimulator through platinum electrodes placed alongside the bath (0.5 Hz, bipolar pulses with voltages 50% above myocyte voltage threshold). Changes in cell length during shortening and re-lengthening were captured for 10 s (30 traces for each cell) and analyzed using soft edge software (IonOptix).

### Echocardiography

 Mice were anesthetized with direct inhalation of isoflurane (1.5-2%). The chest hair was removed and the mouse was placed on a heated platform (38°C) with paws attached to echocardiograph (ECG) leads. A rectal probe was inserted for core temperature monitoring, which was maintained at 36-37°C with the use of a heating lamp and a heated platform. Continuous ECG tracing was recorded throughout the echocardiography study. Echocardiographic imaging was obtained using a VisualSonics Vevo 2100 Imaging System (Toronto, Canada) with an MS400 (30 MHz centerline frequency) probe as previously described [24]. Briefly, parasternal long axis (PSLAX) views in B-mode were obtained. The B-mode guided M-mode view at the papillary muscle level was obtained for the evaluation of end-diastolic and end-systolic left ventricular wall and chamber dimensions in the PSLAX view while the B-mode guided M-mode view at the aortic root was obtained for aortic valve function and root measurements. Color Doppler (CD) of the left ventricular outflow tract was obtained by selecting a relevant region of interest. Small lateral movements were performed through direct and careful study to visualize the presence of aortic insufficiency. When it was visualized, the image was obtained together with Pulse wave Doppler (PD) of the jet. Using the PSLAX M-mode, the left ventricular cavity size and wall thickness was measured. The ejection fraction (EF), fractional shortening (FS), left ventricular end diastolic volume (LVVD) and left ventricular end systolic volume (LVVS) were calculated as previously described [25]. Velocity vector imaging (VVI) derived parameters that measure volume independent myocardial function included global circumferential strain and global radial displacement. These were measured using VevoStrain software (Vevo 2100, v1.1.1 B1455, Visualsonic, Toronto) and were calculated from the short axis images using calculations based on the finite deformation therapy as based on the change in length of a segment divided by its original length [26]. 

### Blood Pressure Telemetry

 Radio-telemetry transmitters (Data Sciences International, St. Paul, MN) were placed into the distal left common carotid and positioned subcutaneously in the intrascapular region of the back with adhesive into mice anesthetized with inhaled isofluorane. Mice were maintained on Buprinorphine at 0.1 mg/kg post operatively with intraperitoneal injections every 12 hr for 2 days. Study mice were monitored daily for changes in their health; moribund mice were euthanized using carbon dioxide asphyxiation. Following a 4 week recovery and acclimation period, blood pressure and heart rate measurements were collected from conscious mice at 10 sec intervals during the 12 hr daylight period and averaged using A.R.T. Platinum Software (Data Sciences International). Prior to captopril delivery, daily water consumption was measured and captopril was delivered via the water at a dose of 40 mg/kg/day. Mice were allowed to equilibrate to captopril administration and withdrawal for 24 hr before blood pressure data was collected. Data are presented as daily averages of 10-sec interval recordings during the daylight period.

### Histological and Immunohistological Analyses

 Hearts and aortas were collected from carbon dioxide-asphyxiated mice. Formalin-fixed paraffin embedded hearts (10 per genotype) were cut on the short axis mid papillary into 4 µm sections and stained with hematoxylin and eosin (H&E), Masson’s Trichrome or Verhoeff-Van Geison stains and analyzed for irregular morphology and fibrosis. Cardiomyocyte size was measured using ImageJ 1.44P analysis software on Masson’s trichrome stained sections at 400x magnification. Thoracic aortas were collected from saline-perfused mice and fixed in formalin. Five micrometer paraffin-sections of ascending aortas were stained in Masson’s trichrome or with a rabbit antibody against CTGF, 1:500 dilution, (Abcam, ab6992) followed by detection using immunoperoxidase reagents (Vector) and counterstained with hematoxylin. Images were processed with linear contrast and brightness adjustments using Photoshop CS5.

### Cell and Tissue Immunoblotting

Tissues isolated from carbon dioxide-euthanized mice were homogenized in cold 0.2 μm-filtered RIPA buffer containing 0.05 mol/L Tris-HCl pH 7.4, 0.15 mol/L NaCl, 0.001 mol/L EDTA, 0.5% Na-deoxycholate, 0.5% NP-40 and 0.1% SDS containing protease and phosphatase inhibitor cocktails (Roche, Complete protease inhibitor and PhosSTOP phosphatase inhibitor tablets) plus 1 mmol/L PMSF. Tissues were homogenized in ground glass mortar and pestle, sonicated 10 pulses at 50% output then cleared by centrifugation at 10,000 x g for 15 min at 4°C. Tissue and cellular proteins (40 µg per lane) were resolved on a 4-12% SDS-polyacrylamide gel and then transferred to PVDF blotting membranes. Non-specific sites on the membranes were blocked by incubation for 1 hr in Tris-buffered saline containing 0.1% Tween 20 and 5% non-fat dry milk. The membranes were incubated with a rabbit anti-mouse Lrp1 antibody [27] or rabbit anti-mouse CTGF antibody followed by horseradish peroxidase-conjugated secondary antibody then visualized by chemiluminescence after exposure to Kodak Biomax films. Equal loading of protein samples was determined using a GAPDH antibody (Cell Signaling, 2118) or anti-α-tubulin antibody (ThermoFisher, MS-581-P1) and densitometry using gel analysis tools in ImageJ.

### Statistical Analysis

Data are reported as mean ± SEM. Statistical comparison between groups was made using ANOVA and the Holm-Sidak method for multiple pair-wise comparisons followed by a 2-tailed Student’s *t* test to evaluate levels of significance at 95% confidence. For incidence of aortic insufficiency, Kaplan Meier survival curves were performed. Heart weight to body weight values were analyzed using Spearman correlation and linear regression analysis. Differences were deemed significant at *P* < 0.05 using SigmaPlot ver. 11 statistical analysis software.

### Ethics Statement

 This study was carried out in strict accordance with the recommendations in the Guide for the Care and Use of Laboratory Animals of the National Institutes of Health (Public Health Service Assurance on Humane Care of Laboratory Animals number A3295-01). The protocol was reviewed and approved by the Institutional Animal Use and Care Committee of the University of Cincinnati.

## Results

### Cardiovascular Deletion of Lrp1 and Cardiomyocyte Mechanics

**Figure 1:**
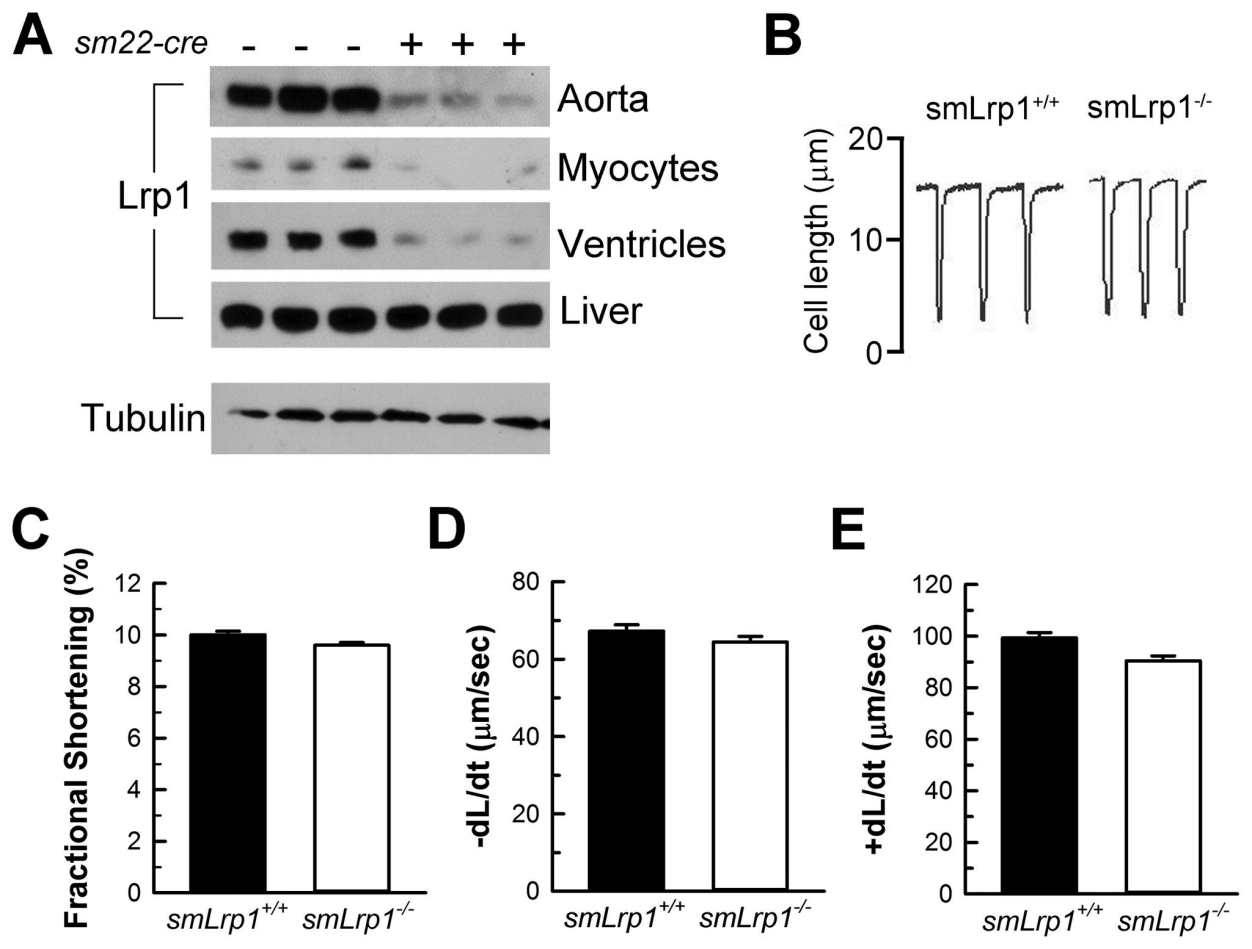
Lrp1 disruption in cardiovascular tissues and isolated cardiac myocyte function. **A**, Immunoblots of Lrp1 in tissue and cell extracts from heart, aorta, liver and cardiomyocytes demonstrated disruption of Lrp1 protein in cardiovascular tissues of *sm22-cre* Lrp1^*flox/flox*^ mice. Cardiomyoctye mechanics were demonstrated in (**B**) representative cell shortening tracings of smLrp1^+/+^ and smLrp1^-/-^ cardiomycytes, as well as (**C**) fractional shortening, (**D**) rates of relaxation, -d*L*/d*t*, and (**E**) rates of contraction, +d*L*/d*t*. n = 3 mice per group for Lrp1 immunoblots and n = 27 cells from 3 smLrp1^-/-^ hearts; n = 20 cells from 2 smLrp1^+/+^ hearts. Data are mean ± SEM.

 Immunoblots of aortic and heart tissues showed sm22 *cre*-mediated recombination in homozygous *Lrp1* ^*fl/fl*^ mice depletes Lrp1 expression in vascular smooth muscle cells and cardiac myocytes (Figure 1A). Examination of mechanical parameters of cardiomyocytes isolated from *smLrp1* ^*+/+*^ and *smLrp1* ^*-/-*^ mice showed no differences in fractional shortening, rates of relaxation, and contraction (Figures 1B-1E).

### Age-dependent Increase in Aortic Root Diameter and Incidence of Aortic Insufficiency with Smooth Muscle Lrp1 Deficiency

**Figure 2:**
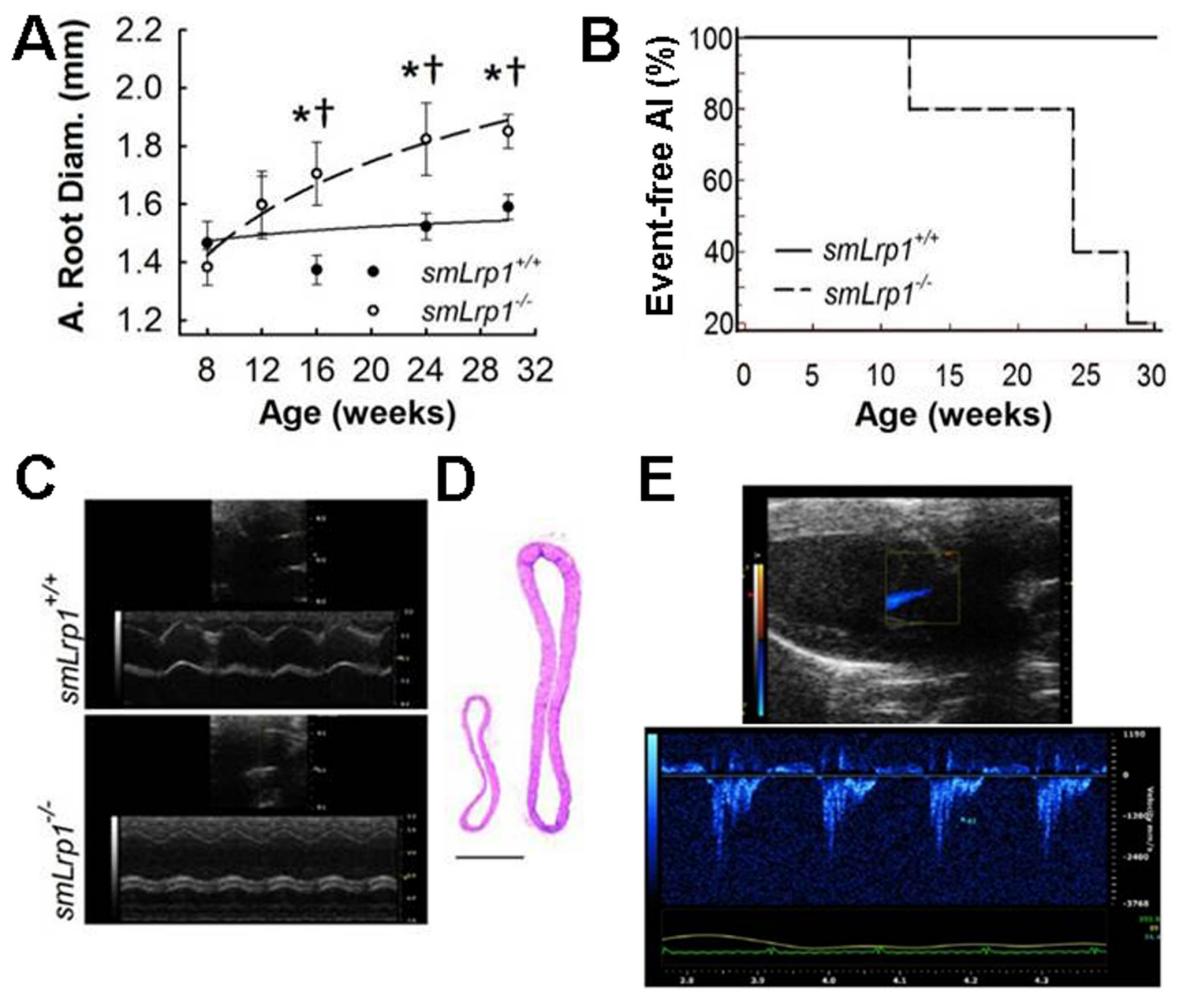
Aortic root diameter and aortic insufficiency. **A**, Changes in aortic root diameter during the 22 week study period were measured with echocardiograms at indicated intervals. *P<0.01, versus aortic root diameter of eight week old *smLrp1* ^*-/-*^ mice; †*P*<0.05, pair wise comparison among age-matched *smLrp1* ^*+/+*^ mice. **B**, Kaplan Meier curve analysis of aortic insufficiency events in *smLrp1* ^*-/-*^ and *smLrp1* ^*+/+*^ littermates (*P*=0.05). n = 6 mice per genotype. **C**, Representative 2D image of aortic root with M-mode imaging at 16 weeks of *smLrp1* ^*-/-*^ (bottom) and wildtype littermate (top). **D**, Hematoxylin-eosin-stained, cross-section of ascending aorta at ~3mm from aortic valve from 40-week old *smLrp1* ^*+/+*^ (left) and *smLrp1* ^*-/-*^ (right) Scale bar, 500μm. **E**, Detection of AI jet (blue) in 2D long axis image with color Doppler on a 24 week old *smLrp1* ^*-/-*^ mouse with the same AI jet projected along pulsed Doppler M-mode.

 Initial echocardiogram measurements showed no difference in aortic root sizes between *smLrp1* ^*+/+*^ and *smLrp1* ^*-/-*^ littermates when the animals were 8 weeks old. In contrast, significant dilation was observed in the aortic roots of *smLrp1* ^*-/-*^ mice compared to *smLrp1* ^*+/+*^ mice beginning at 16 weeks of age (1.37 ± 0.05 mm vs.1.7 ± 0.1 mm; *P*<0.01) and progressing through the final measurement at 30 weeks (1.59 ± 0.04 mm vs. 1.85 ± 0.05 mm; *P*<0.001), resulting in significant age-dependent differences in aortic root diameter (*P*<0.05)(Figure 2A, 2C). Additionally, while all animals survived throughout the study and no aortic insufficiency was observed in *smLrp1* ^*+/+*^ mice as they age, the *smLrp1* ^*-/-*^ mice showed age-dependent increase in incidence of aortic insufficiency (Figure 2B). Hematoxylin and eosin stained cross-sections of the ascending aorta of 40-week old mice demonstrated the extent of dilation and medial thickening observed in *smLrp1* ^*-/-*^ mice (Figure 2D). Additionally, serial color Doppler and pulse wave Doppler measurements of the left ventricular outflow tract revealed aortic insufficiency in all of the 30-week old *smLrp1* ^*-/-*^ mice examined, while none of the *smLrp1* ^*+/+*^ littermates displayed any aortic insufficiency (Figure 1B, 2E).

### Increased Pulse Pressure in Lrp1-deficiency

 A separate group of mice were subjected to invasive blood pressure monitoring. Blood pressure telemetry using intra-carotid probes in 24-week old mice recorded 44% higher pulse pressure in *smLrp1* ^*-/-*^ mice compared to *smLrp1* ^*+/+*^ mice (Table 1). The primary contributor of this phenotype appeared to be a 16% reduction in diastolic pressure in the *smLrp1* ^*-/-*^ mice whereas systolic blood pressure was not different. Captopril treatment reduced systolic and diastolic blood pressure in all mice but significantly reduced pulse pressure in *smLrp1* ^*-/-*^ mice to levels observed in untreated *smLrp1* ^*+/+*^ mice (Table 1).

**Table 1:** Hemodynamic properties of 24 week-old *smLrp1* ^*+/+*^ and *smLrp1* ^*-/-*^ littermates.

		***smLrp1*^*+/+*^** (n=6)	***smLrp1*^*-/-*^ (n=6)**	***P* value**
**Mean Arterial Pressure (mmHg)**	Untreated	112.63 ± 1.51	102.12 ± 2.92	0.123
	Captopril	95.95 ± 2.43	88.50 ± 2.39	0.267
**Systolic Blood Pressure (mmHg)**	Untreated	127.51 ± 2.13	125.39 ± 3.30	0.608
	Captopril	110.31 ± 1.56	105.29 ± 1.45	0.169
**Diastolic Blood Pressure (mmHg)**	Untreated	97.48 ± 2.06	82.27 ± 2.50	0.023
	Captopril	77.74 ± 0.97*	73.84 ± 2.97	0.001
**Pulse Pressure (mmHg)**	Untreated	29.99 ± 2.73	43.18 ± 2.04	0.001
	Captopril	28.09 ± 2.73	31.72 ± 1.83*	0.025
**Heart Rate (bpm)**	Untreated	574.61 ± 7.07	589.47 ± 15.97	0.427
	Captopril	592.15 ± 5.37	616.41 ± 6.90	0.032

Data represent mean±SEM; *P* value column represents genotypic comparisons within treatments

* *P*<0.001, versus untreated group within each genotype.

### Impaired Cardiac Functions in Aged smLrp1^-/-^ Mice

**Figure 3:**
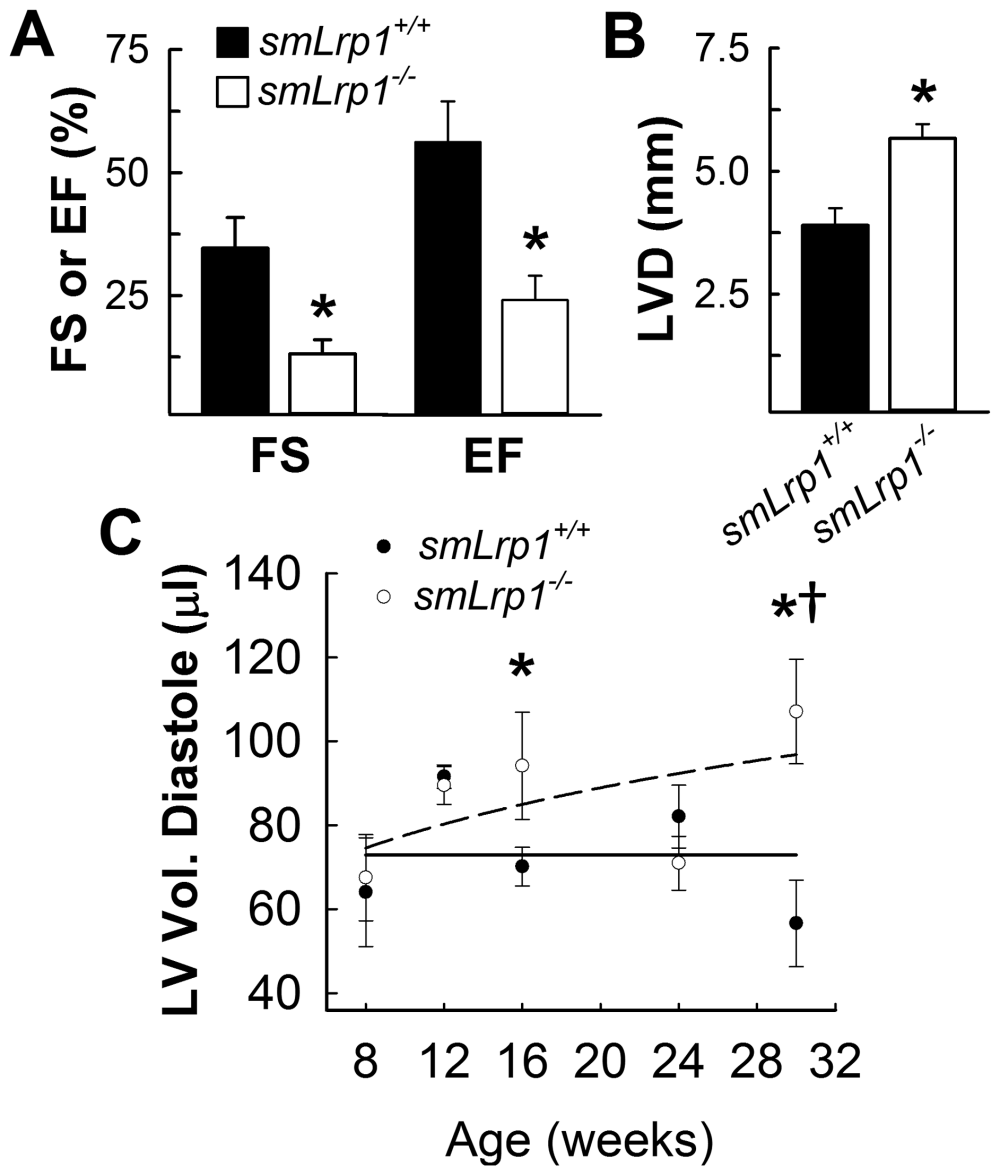
Cardiac output and left ventricular dilation. **A**, Fractional Shortening (FS), Ejection Fraction (EF) and (**B**) Left ventricular diameter in diastole (LVVD) measurements collected from 36 week old *smLrp1* ^*+/+*^ and *smLrp1* ^*-/-*^ mice. * *P*<0.05 **C**, Left ventricular volumes during diastole were determined during the 22 week study period with echocardiograms at indicated intervals. *P<0.01, versus aortic root diameter of eight week old *smLrp1* ^*-/-*^ mice; †*P*<0.05, pair wise comparison among age-matched *smLrp1* ^*+/+*^ mice.

 Twelve-week old animals were examined via echocardiography and showed no significant differences in systolic function (EF and cavity dimensions) as well as VVI derived displacement (0.36 ± 0.06 mm vs. 0.46 ± 0.1 mm; *P* = 0.41) and strain measurements (-18.5 ± 3.9 vs. -22.95 ± 5.0; *P* = 0.49). However, echocardiographic analysis of a cohort of 36-week old animals demonstrated significant reduction in systolic function in *smLrp1* ^*-/-*^ mice. Specifically, a 38% reduction in fractional shortening, 43% reduction in ejection fraction, and 68% increase in left ventricular diameter (LVVD) were observed in *smLrp1* ^*-/-*^ mice compared to *smLrp1* ^*+/+*^ mice (Figure 3A, 3B). Additional experiments designed to assess age-dependent remodeling of left ventricular cavity were performed using age-matched cohorts beginning at 8-weeks of age, with measurements taken every 4 weeks for 32 weeks. Left ventricular diastolic volume was found to increase with age in *smLrp1* ^*-/-*^ mice, compared to *smLrp1* ^*+/+*^ mice which displayed similar LV volume throughout this period (Figure 3C). However, when the animals reached 16 weeks of age, a strong trend towards LV cavity dilation was observed in the *smLrp1* ^*-/-*^ mice in comparison to the 12-week old mice (*P* < 0.01) and to their age matched *smLrp1* ^*+/+*^ littermates (*P* < 0.05). Severe dilation was detected in the *smLrp1* ^*-/-*^ mice at 30 weeks of age (LV vol d 107 ± 12.4 µl vs. 56.6 ± 10.3 µl; *P* < 0.01).

### Cardiac Enlargement and Dilated Ascending Aorta in Aged smLrp1^-/-^ Mice

**Figure 4:**
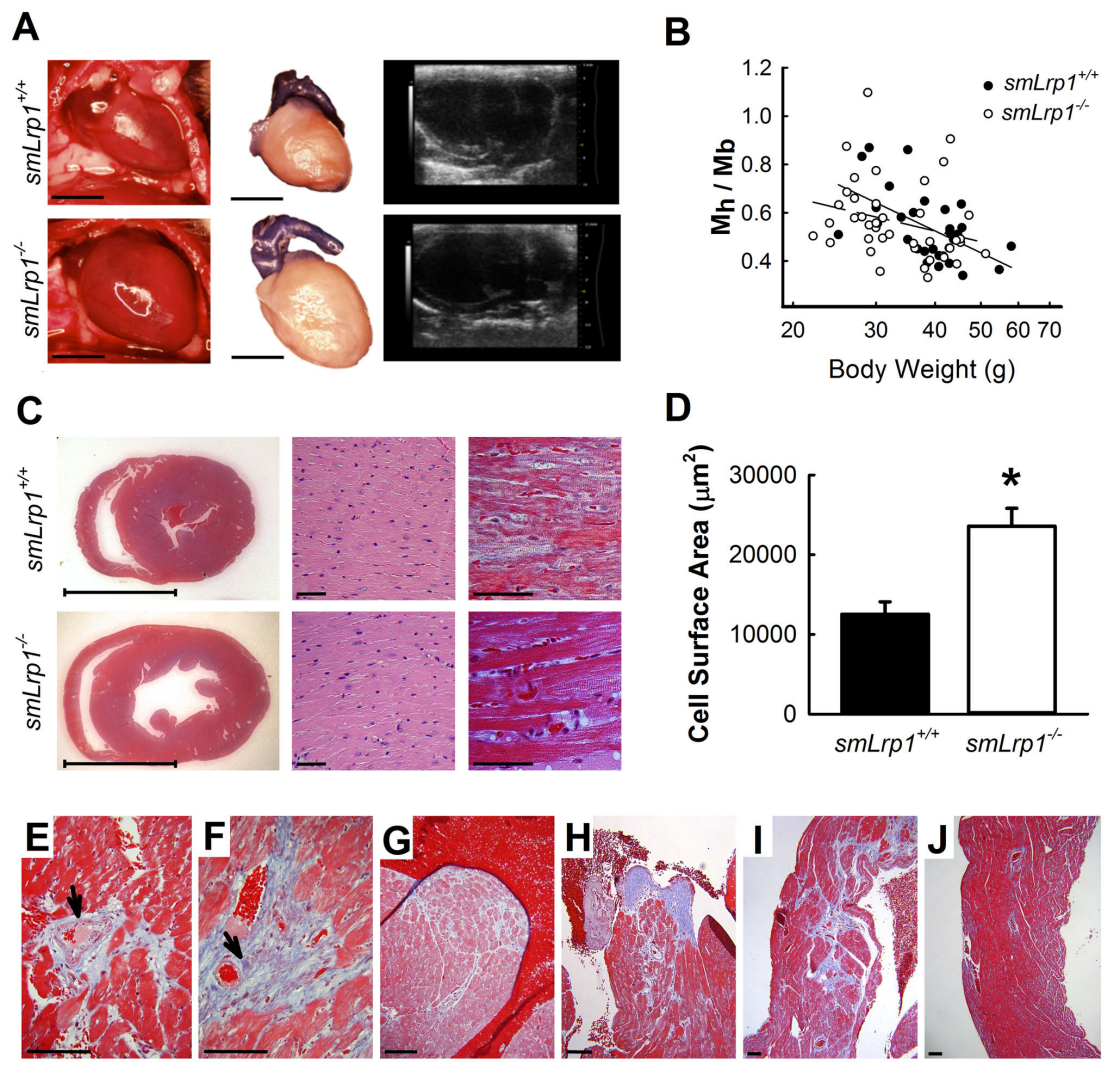
Heart defects in *smLrp1*-deficient mice. **A**, Ventral view of intact heart from 40 week old mice, formalin-fixed dissected heart and parasternal long axis view echocardiogram, scale bar=5 mm. **B**, Heart mass (M_h_) to body mass (M_b_) ratios among 24 *smLrp1* ^*+/+*^ (solid line) and 42 *smLrp1* ^*-/-*^ (dashed line) mice across the experimental age spectrum from 29 *smLrp1* ^*+/+*^ mice and 42 *smLrp1* ^*-/-*^ mice. (*Spearman* *Correlation*: r_s_ -0.489 and *P* 0.0074 for *smLrp1* ^*+/+*^ and r_s_ -0.402 and *P* 0.0086 for *smLrp1* ^*-/-*^; *Regression* *Analysis*: Slope -0.933 for smLrp1^+/+^ and -0.461 for *smLrp1* ^*-/-*^). **C**, Mason’s trichrome-stained short axis sections from 40 week old mice, left panels (bar = 5mm); hematoxylin and eosin and Mason’s trichrome staining of parenchymal area of left ventricle (LV), center and right panels, respectively (bar = 30 μm). **D**, Longitudinal cell surface area of LV cardiomyocytes. **E**-**I**, Perivascular fibrosis and interstitial fibrosis (Mason’s trichrome, blue area) observed in intramural coronary arteries of LV free wall; arrows indicate arteries (**E**, **F**), papillary muscle (**G**, **H**) and right ventricle parenchyma and coronary arteries of hearts from *smLrp1* ^*-/-*^ (**I**) and *smLrp1* ^*+/+*^ mice (**J**) (bar = 30 μm). * *P* < 0.05.

 Gross and echocardiographic analysis of heart morphology revealed increases in heart size and dilated ascending aortas in *smLrp1* ^*-/-*^ mice that were >36 weeks of age (Figure 4A). As expected, heart weight to body weight ratios were found to be higher in *smLrp1* ^*-/-*^ mice compared to *smLrp1* ^*+/+*^ mice throughout the age range from 16 to 70 weeks of age (Figure 4B). Assessment of tissue pathology in histological sections of 48-week old mouse hearts revealed LV enlargement (Figure 4C) and a significant increase in myocyte longitudinal area in parenchymal regions of the LV free wall (Figure 4D). The direct assessment of tissue fibrosis with Masson’s Trichrome stain in cardiac sections demonstrated increased perivascular fibrosis with outward extension of fibrotic lesions surrounding intramural coronary arteries of *smLrp1* ^*-/-*^ mice (Figure 4E, F). Differences in pericellular fibrosis were not detected between *smLrp1* ^*+/+*^ and *smLrp1* ^*-/-*^ mice in the parenchymal regions, except for extensive fibrosis observed in LV papillary muscles of *smLrp1* ^*-/-*^ mice (Figure 4G, H). Finally, whereas extensive and continuous fibrotic lesion tracts extending outward from coronary arteries were observed sporadically in ventricle walls of *smLrp1* ^*-/-*^ mice (Figure 4I), these fibrotic lesion tracts were not observed in *smLrp1* ^*+/+*^ mice (Figure 4J).

### Abnormal Aorta and Coronary Artery Structure in smLrp1^-/-^ Mice

**Figure 5:**
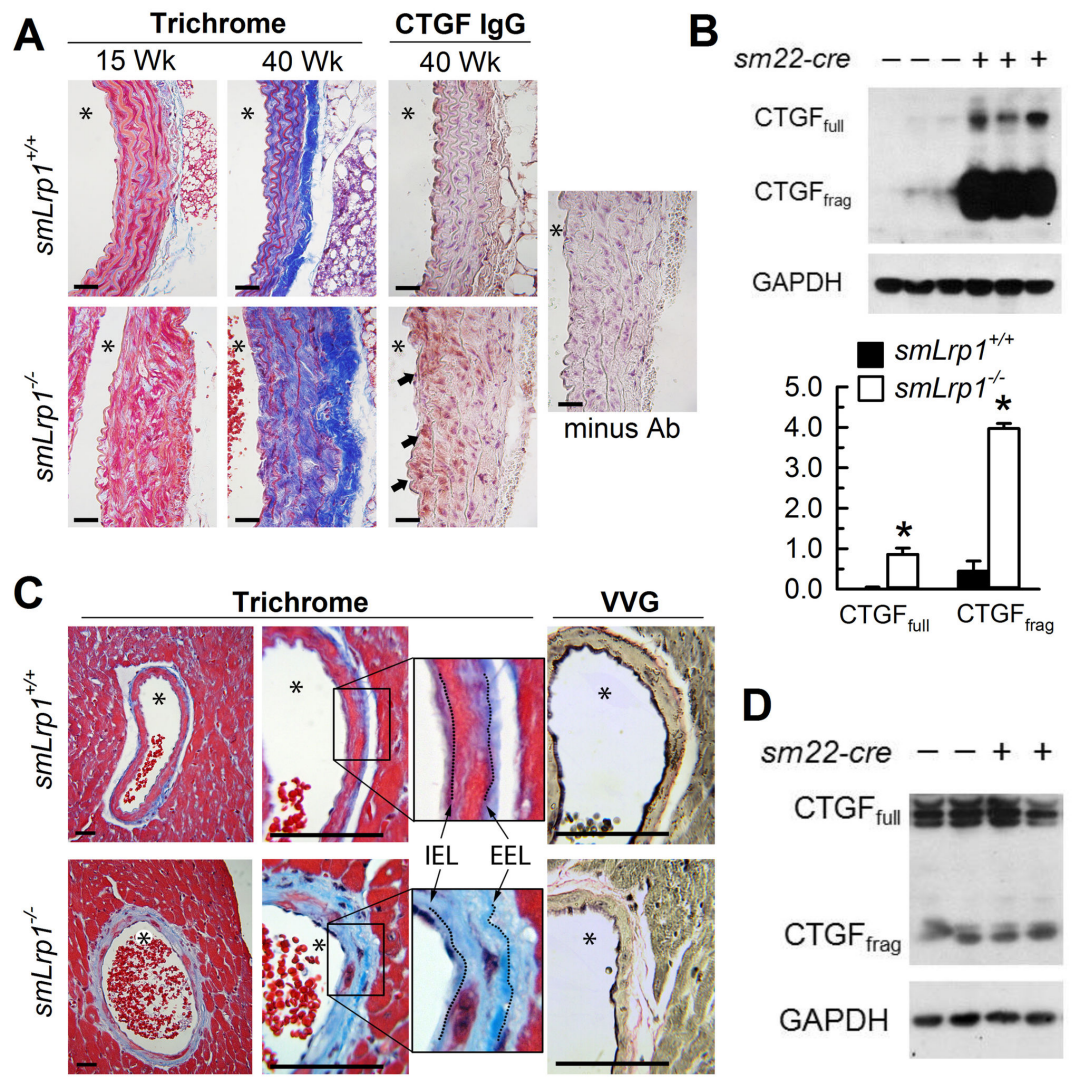
Aorta and coronary artery fibrosis and vascular CTGF accumulation. **A**, Mason’s Trichrome staining of ascending aortas of 15 and 40 week old mice and immunostaining with anti-CTGF antibodies (brown staining at arrows) or isotype control antibody and hematoxylin counterstained nuclei. Solid arrows indicate regions with abundant CTGF-positive signal, asterisk denotes vessel lumen (bar = 30 μm). **B**, Immunoblots of tissue CTGF full length and hydrolytic fragment content in aortas sampled from 30 week old mice. Bar graphs represent densitometric means ± SEM of CTGF (full length and fragment) normalized to GAPDH from three *smLrp1* ^*+/+*^ and three *smLrp1* ^*-/-*^ tissue preparations. * *P*<0.05 between genotypes **C**, Mason’s trichrome staining of LV coronary arteries and surrounding myocytes (fibrosis, blue area; asterisk denotes vessel lumen) with inset showing internal (IEL) and external (EEL) elastic laminae; Vanhoeff-Van Geison (VVG) staining of internal and external elastic laminae (black colored lines) (bar = 30 μm). **D**, Tissue expression of CTGF in hearts collected from 30 week old mice.

Ascending aortas from smLrp1^-/-^ mice were consistently thickened and characterized by increased cell nuclei, fractured elastic laminae and accumulation of extracellular matrix (Figure 5A). These differences increased as the mice age (15 week versus 30 week) with continued intra-lamellar thickening, extracellular matrix deposition and cellular disorganization. Increased cellular and pericellular accumulation of CTGF was also noted in the medial layers (Figure 5A). Immunoblotting of aorta tissue homogenates demonstrated significantly accumulation of both 38-kDa full-length CTGF and 18-20-kDa CTGF proteolytic fragments in *smLrp1* ^*-/-*^ mice (Figure 5B). 

Coronary arteries were examined for differences in structure and tissue composition after staining with Masson’s Trichrome and Verhoeff-Van Geison (VVG) stains. Analysis of coronary arteries within the left ventricle demonstrated an accumulation of collagen within the tunica media and adventitia of *smLrp1* ^*-/-*^ mice (Figure 5C). Staining of elastic lamina with VVG indicated continuous internal and external lamellae, and morphometric analysis revealed an absence of vessel remodeling, without changes in luminal area or medial thickness or evidence of occlusions in either *smLrp1* ^*+/+*^ of *smLrp1* ^*-/-*^ mice (Figure 5C). Interestingly, abundant cell and collagen deposition in the adventitia regions of coronary arteries which occasionally extended outward into cardiac muscle tissues were observed in *smLrp1* ^*-/-*^ mice (Figure 5C). Moreover, while CTGF was detectable in heart tissues by immunoblots there were no differences in CTGF levels in the heart tissues of *smLrp1* ^*-/-*^ and *smLrp1* ^*+/+*^ mice (Figure 5D).

### Deletion of Smooth Muscle Lrp1 Increases ERK1/2 and SMAD2 Phosphorylation

**Figure 6:**
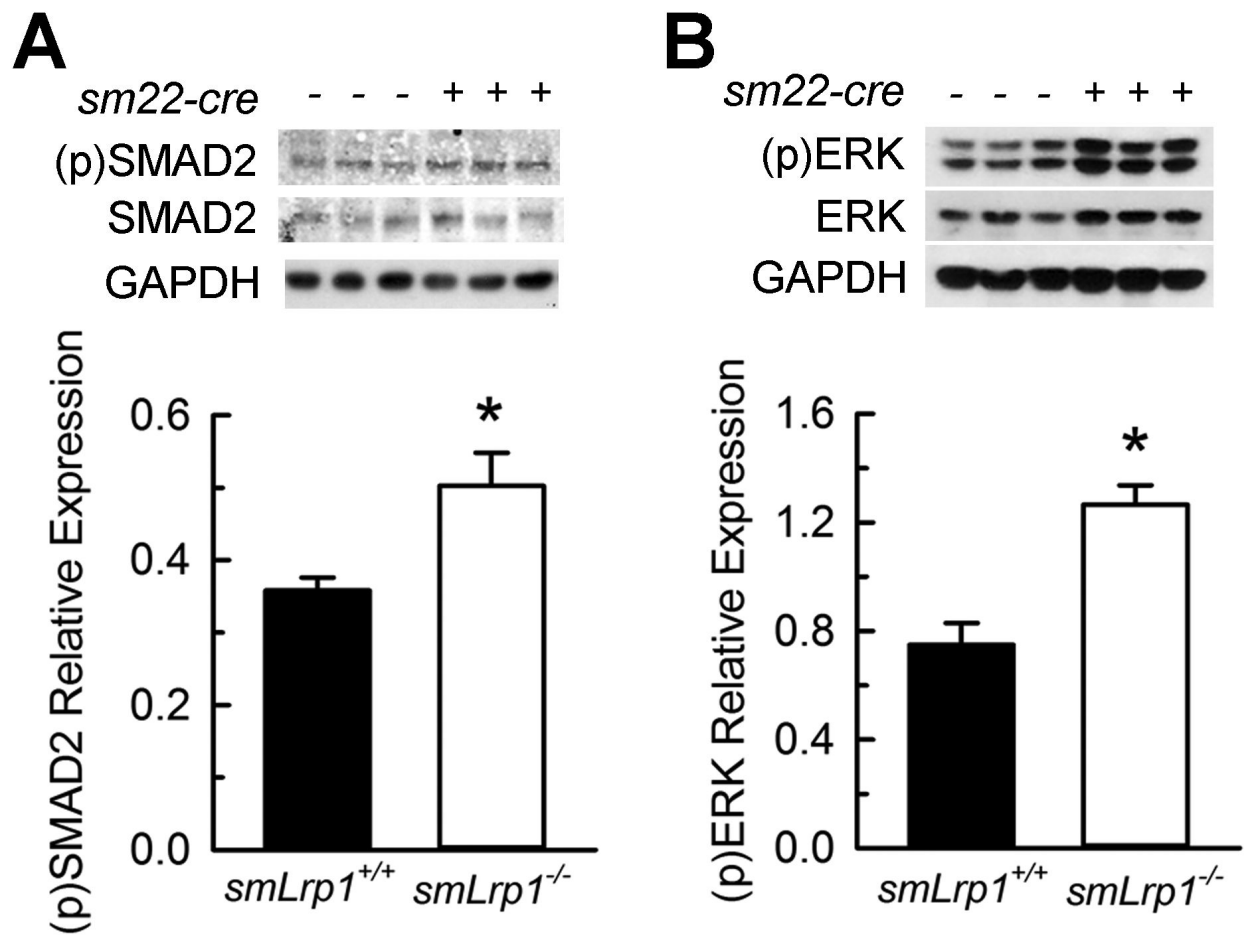
ERK and SMAD2 signaling in thoracic aortas. **A**, Immunoblots of tissue (p)SMAD2 and total SMAD2 detected in thoracic aortas sampled from 30 week old mice; 3 mice per genotype. Bar graphs represent densitometric means ± SEM following normalization to GAPDH. **B**, Tissue expression of (p)ERK1/2 and ERK2 in thoracic aortas from 30 week old mice. * *P*<0.05 between genotypes.

 Because CTGF is well characterized as a TGF-β response gene, thoracic aorta protein extracts were assayed for critical TGF-β cellular signaling molecules. Aortas from 30 week old *smLrp1* ^*-/-*^ mice demonstrated increased abundance (28%) of phosphorylated SMAD2 protein (Figure 6A) compared to *smLrp1* ^*+/+*^ mice. In addition, phosphorylated ERK1/2 were significantly increased (38%) in aortic extracts from *smLrp1* ^*-/-*^ mice (Figure 6B). 

## Discussion

This study demonstrated that cardiovascular deficiency of Lrp1 causes vascular changes resulting in aortic dilation and insufficiency and development of dilated cardiomyopathy. Using echocardiography, our longitudinal studies of *smLrp1* ^*+/+*^ and *smLrp1* ^*-/-*^ mice revealed an age-dependent process in which mice younger than 16 weeks of age had normal cardiac function. Aortic root dilation in *smLrp1* ^*-/-*^ mice began at 16 to 20 weeks of age, followed by the development of aortic insufficiency that ultimately coincided with or contributed to left ventricular dilation and impaired function. Our assessment of cardiomyocyte mechanical parameters in mice 12 weeks of age suggested there were no functional abnormalities of heart muscle cells prior to onset of aortic insufficiency and correlated with normal cardiac function observed by echocardiography in mice of similar age. These results are consistent with previous reports documenting smooth muscle Lrp1 deficiency phenocopies Marfan-like syndromes [17]. Importantly, when put into context of the findings reported by Meijboom et al [28] and others [29,30] in patients with Marfan syndrome, these results suggest that most of the cardiac pathology associated with Marfan and Marfan-like syndromes may be secondary to vascular pathologies and not a primary cardiac defect.

Similar to cardiovascular pathologies observed in Marfan syndrome, medial layers of the aortas of smooth muscle Lrp1-deficient mice show extensive abnormalities including fragmentation, disorganization and loss of elastic laminae and accumulation of matrix proteins. The overall effect is progressive medial thickening with aortic root dilation and increase in pulse pressure secondary to reduced diastolic blood pressure. These results are in striking contrast to a recent study showing reduced diastolic blood pressure as well as systolic and mean arterial pressure in *smLrp1* ^*-/-*^ mice [18]. The discrepancy between the two studies may reflect differences in the age of the mice used for experiments as well as the method of data collection. The Muratoglu study examined blood pressure using intra-aortic catheter in anesthetized mice at 16 weeks of age, whereas our study measured blood pressure with intra-carotid cannula in conscious 30-week old mice. The use of anesthetics has been shown previously to reduce cardiac output by suppressing heart rate or reducing stroke volume, thereby reducing systolic blood pressure [31]. Indeed, heart rates were reported to be moderately lower in anesthetized *smLrp1* ^*-/-*^ mice [18], and we observed moderately increased heart rates in conscious *smLrp1* ^*-/-*^ mice in restoring cardiac output and systolic blood pressure. In any event, the compromised vascular reactivity of aortic rings isolated from *smLrp1* ^*-/-*^ mice reported previously is supportive of a stiffened aortic phenotype [16]. 

Vascular pathology associated with Marfan-like syndromes is characterized by abnormal homeostasis of the extracellular matrix manifested by elastic fiber disruption, incompetent matrix architecture and/or dysregulation of TGF-β activation of signaling [32]. Previous studies demonstrated an atheroprotective role of vascular Lrp1 in homozygous LDL receptor knockout mice while showing that Lrp1 expression can function to regulate TGF-β signaling in smooth muscle cells and maintain vessel wall integrity [17]. Consistent with these findings, we observed increased SMAD and ERK signaling in *smLrp1* ^*-/-*^ mice, which can also contribute to aortic aneurysm in mice [33]. However, elevated SMAD phosphorylation was not observed in another study [18]. The difference in results from the two studies remains unknown but may possibly be due to differences in age of the animals examined. Regardless, both studies revealed dramatic increase of vascular CTGF bioavailability in the absence of Lrp1. Therefore, the vascular phenotype observed in *smLrp1* ^*-/-*^ mice is likely due to deregulation of TGFβ signaling [34], compounded by impaired receptor-mediated endocytosis of the potent fibrotic mediator CTGF.

Histopathology of the myopathies observed in *smLrp1* ^*-/-*^ mice was characterized by myocyte hypertrophy and tissue fibrosis of perivascular and papillary muscle tissues. These observations are consistent with transgenic mouse models of hypertrophic myopathies and have been shown to be mediated by non-myocyte activation via TGF-β signaling [35]. Interestingly, differences in pericellular fibrosis were not detected in parenchymal regions of the left ventricle and the presence of fibrosis in both genotypes could reflect an aging-related process [36]. Collagen accumulation in coronary arteries of *smLrp1* ^*-/-*^ mice is also consistent with the changes observed in conduit arteries in the same mice but without the severe disruption of elastic layers, smooth muscle hyperplasia, and vessel remodeling. Excess collagen deposition in the tunica media and adventitia could result in increased coronary artery stiffness with age, which may be exacerbated by ventricular hypertrophy resulting from aortic insufficiency [37]. These changes in coronary compliance could alter local hemodynamic conditions and impair cardiac tissue perfusion, but is unlikely due to the lack of apoptosis noted in these areas. Importantly, similar to our observations in *smLrp1* ^*-/-*^ mice, large artery stiffness has been demonstrated to reduce coronary flow reserve and impair cardiac perfusion [38], while long term angiotensin-converting enzyme inhibition can improve coronary flow reserve in a hypertensive setting [39]. 

The hemodynamic responses observed with captopril in 24-week old *smLrp1* ^*-/-*^ mice suggest that the resistance vasculature responded appropriately to RAAS blockade, and the normalization in pulse pressure is consistent with a beneficial effect on cardiac and vascular function in the setting of aortic insufficiency. Previous studies have shown that RAAS blockade lowers disastolic pressure in hypertensive patients [40] and improves hemodynamics in mouse models of Marfan syndrome via inhibition of TGFβ signaling and prevention of elastic fiber fragmentation [41]. These findings are also consistent with the clinical findings of RAAS blockade in Marfan syndrome [20,21]. Taken together, these results showed that smooth muscle Lrp1 deficiency phenocopies vascular abnormalities of Marfan-like syndromes, in a mechanism related to aberrant TGFβ signaling and Smad2 phosphorylation that can be suppressed by RAAS therapy. While a majority of Marfan syndrome can be attributed to mutations in the fibrillin-1 gene, our results documenting Lrp1 inactivation causes similar pathology including the progression to cardiomyopathy suggest that Lrp1 dysfunction may account for at least some of the Marfan-like syndrome unrelated to fibrillin mutations [42]. Additional population studies evaluating a potential relationship between human LRP1 polymorphism with Marfan-like syndrome are warranted. In this regard, association between human LRP1 polymorphism and abdominal aortic aneurysm, another vascular disease associated with Marfan syndrome and aberrant TGFβ signaling [43], has already been noted in the literature [44]. 

 On a technical front, the noninvasive assessment of AI in a mouse model is challenging due to the small size of the heart and the aorta and very fast heart rate [45]. The studies that have evaluated the aorta in mouse models revealed that the ascending aorta [46], the aortic arch [47], and the abdominal aorta [48] could be measured with currently available ultrasound technology. Other studies have used ultrasound to study the aortic valve itself including its development [49] and insufficiency [5]. To the best of our knowledge, this technology has yet to be used to evaluate the progression of aortic insufficiency and its relationship to changes in left ventricular size and function. Our findings may have significant implications in the study of valvular cardiomyopathy. We have confirmed in this model that (i) AI develops within a very narrow time period (16 to 20 weeks), (ii) the subsequent volume overload state leads to cardiomyopathy, and (iii) the animals responded appropriately to afterload reduction. Thus, the *smLrp1* ^*-/-*^ mice may serve as a useful model to test the effects of pharmacotherapy on the development and progression of valvular cardiomyopathy.
